# Therapeutic clothing use in atopic dermatitis in the Netherlands: A population based cohort study

**DOI:** 10.1002/ski2.303

**Published:** 2023-10-17

**Authors:** Aviël Ragamin, Renske Schappin, Suzanne G. M. A. Pasmans, Marie L. A. Schuttelaar

**Affiliations:** ^1^ Department of Dermatology Erasmus MC University Medical Center Rotterdam The Netherlands; ^2^ Department of Dermatology Center of Pediatric Dermatology Sophia Children's Hospital Erasmus MC University Medical Center Rotterdam‐Sophia Children's Hospital Rotterdam The Netherlands; ^3^ Department of Dermatology University Medical Center Groningen University of Groningen Groningen The Netherlands

## Abstract

National prescription data for therapeutic clothing in atopic dermatitis was analysed to investigate the role of therapeutic clothing in atopic dermatitis. Therapeutic clothing is most frequently prescribed by dermatologists in a hospital setting. Most patients only receive one prescription of therapeutic clothing, suggesting a limited role for therapeutic clothing in the long‐term management of atopic dermatitis.

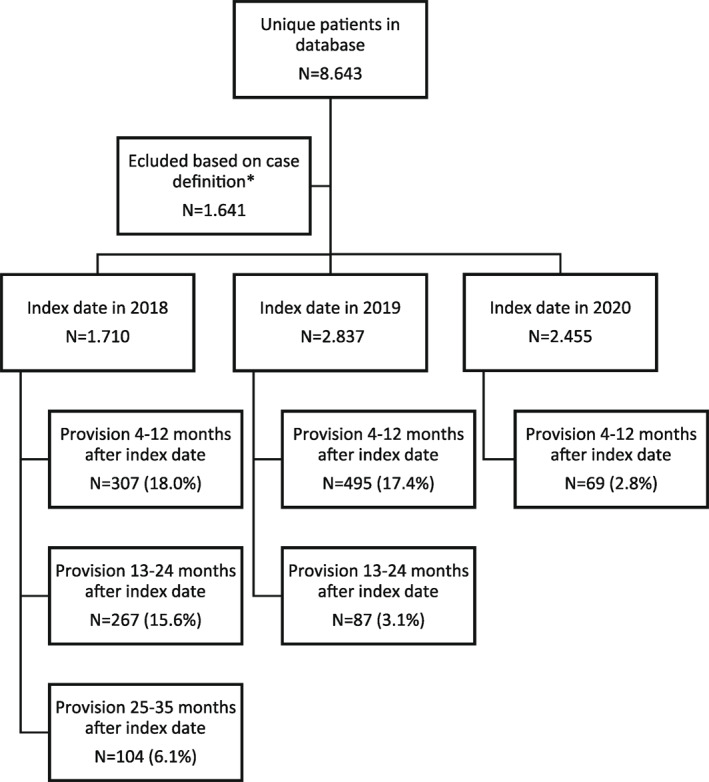

Dear Editor,

Atopic dermatitis (AD) is an inflammatory skin disorder affecting many children and adults.^1^ Although pharmacological treatment options are available, many patients prefer non‐pharmacological (i.e. steroid‐sparring) treatments.^2^ Special clothing, garments, and textiles have been used for decades in AD.^3^ Therapeutic clothing aims to reduce AD symptoms by regulating skin temperature and humidity, protecting against scratching and irritating factors, and fixating ointments to the skin.^4^ Therapeutic clothing has been evaluated in few studies, which generally had small samples and were of short duration, limiting our understanding.^5^, ^6^

In the Netherlands, therapeutic clothing is recommended by the Dutch Society for Dermatology and Venereology (NVDV) and reimbursed for moderate‐to‐severe AD as an addition to topical treatment.^5^ This setting provides a unique opportunity to examine therapeutic clothing. We therefore analysed retrospective administrative data from DeclaCare, the only distributor of reimbursed therapeutic clothing (Binamed® made from micro‐modal and lycra and DermaCura® made from TENCEL® and elastane). Prescription data between January 2018 and December 2020 were extracted and analysed. More recent data were not available due to changes in the registration process. To enhance reliability, we only included patients receiving therapeutic vests or leggings, since therapeutic clothing could also be prescribed for patients with hand eczema only. The date of the first provision during the study period was defined as the index date. The population was stratified in four groups based on age at the index date, infant (age <4 years), child (age ≥4–≤11), adolescent (age ≥12–≤17) and adult (age ≥18). The first provision was defined as all prescriptions between the index date and 3 months of follow‐up, the second provision as provisions between 4 and 12 months after the index date and the third and fourth as provisions between 13–24 and 24–35 months after the index date, respectively. 7002 patients received therapeutic clothing between 2018 and 2021, Figure 1. This accounts for approximately 0.5% of all treated AD patients or 6%–8% of patients treated by dermatologists.^7^, ^8^ Infants comprised the largest group (59.8%), followed by children (24.2%) and adults (15.9%). Most patients were treated in a hospital setting (82.3%) and received therapeutic clothing from a dermatologist (59.5%) or paediatrician (23.8%). Long sleeved therapeutic vests (78.1%) and leggings (70.9%) were prescribed most frequently. Approximately 17.4%–18.0% received a second prescription within 1 year of the index date. For patients with an index date in 2018, further follow‐up was possible. Of these patients, 15.6% received a prescription one‐to‐two years after the index date. Although further follow‐up was limited due to lack of data of 2021, at least 6.1% received a new prescription two to three years after the index date. Overall, we found no major differences in the characteristics between patients who received one prescription and those who received multiple prescriptions. However, compared to patients receiving a single prescription, patients who use therapeutic clothing longer, more often received therapeutic gloves (difference up to 36.6%).

**FIGURE 1:**
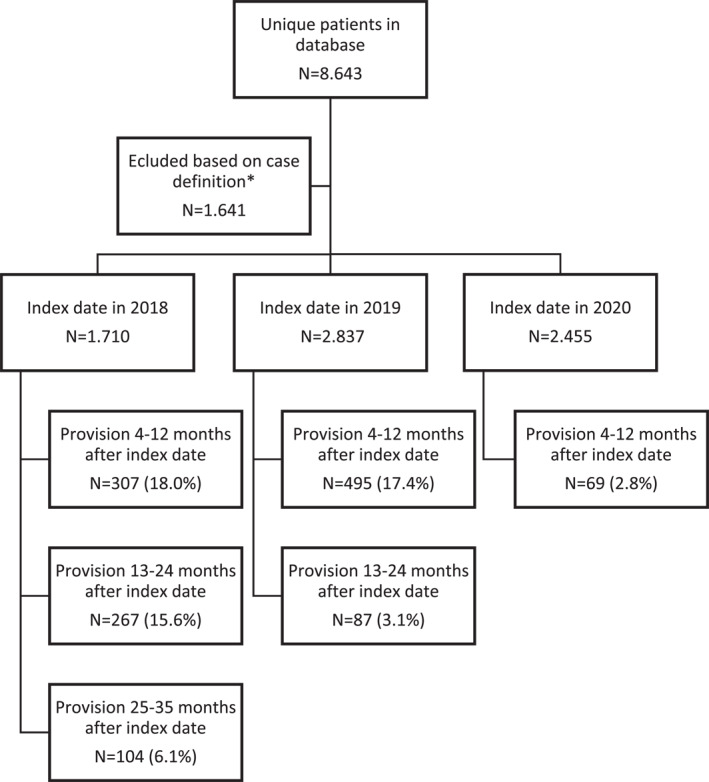
A flowchart of patient inclusion and follow‐up. *Patients were excluded if they did not receive at least one prescription of therapeutic (long or short) sleeved vests or leggings.

Based on these findings, we assume that therapeutic clothing is often used as part of induction therapy during initial consultation to reduce overall disease burden and less often for chronic management of flares and AD symptoms. The underlying reasons are presumably a mix of factors, such as satisfactory response to overall treatment, non‐persisting symptoms, patient preferences, availability of other treatment options, (perceived) lack of effect, and lack of awareness of prescribers on reimbursement regulation. However, based on our data, we found a significant portion of patients who repeatedly received therapeutic clothing. This implies that patients and/or professionals experience added value of therapeutic clothing in a subpopulation of patients. Further investigation of subpopulations would therefore be interesting.

To our knowledge, this is the only study to investigate therapeutic clothing based on national data. A major strength is the access to national prescription data, which enabled us to examine real use of therapeutic clothing. Moreover, we were able to follow up for 3 years, although longer follow‐up would have given greater insight. Important limitations included lack of clinical data and lack of a validated case definition.

To conclude, we found that therapeutic clothing is mostly prescribed for infants with AD in a hospital setting. Most patients do not receive a second prescription, suggesting a limited role for therapeutic clothing in the long‐term management of AD. More research based on clinical data is necessary in order to fully understand the role of therapeutic clothing in the management of AD.

## CONFLICT OF INTEREST STATEMENT

The Department of Dermatology of the Erasmus MC University Medical Centre Rotterdam previously received an unrestricted grant from BAP Medical, D&M B.V., and DeclaCare (part of BENU Netherlands).

## AUTHOR CONTRIBUTIONS

**Aviël Ragamin**: Conceptualisation (equal); Data curation (equal); Formal analysis (equal); Investigation (equal); Methodology (equal); Writing – original draft (equal); Writing – review & editing (equal). **Renske Schappin**: Conceptualisation (equal); Data curation (equal); Formal analysis (equal); Methodology (equal); Supervision (equal); Writing – review & editing (equal). **Suzanne G.M.A. Pasmans**: Conceptualisation (equal); Project administration (equal); Supervision (supporting); Writing – review & editing (supporting). **Marie L.A. Schuttelaar**: Conceptualisation (equal); Investigation (equal); Methodology (equal); Project administration (equal); Supervision (equal); Writing – review & editing (equal).

## FUNDING INFORMATION

This article received no specific grant from any funding agency in the public, commercial, or not‐for‐profit sectors.

## ETHICS STATEMENT

This study was exempt from the Dutch Medical Research Involving Human Subjects Act according to the institutional review board of Erasmus MC (MEC–2023–0199). Furthermore, in accordance with Dutch legislation the need to obtain informed consent was waived for this retrospective observational study with anonymised data.

## Data Availability

The data that support the findings of this study are available from DeclaCare. Restrictions apply to the availability of these data, which were used under license for this study. Data are available from the authors [SP] with the permission of DeclaCare (part of BENU Netherlands).
